# Ethical Issues in Online Psychotherapy: A Narrative Review

**DOI:** 10.3389/fpsyt.2019.00993

**Published:** 2020-02-11

**Authors:** Julia Stoll, Jonas Adrian Müller, Manuel Trachsel

**Affiliations:** Institute of Biomedical Ethics and History of Medicine, University of Zurich, Zurich, Switzerland

**Keywords:** online psychotherapy, telepsychology, telepsychiatry, ethics, technology

## Abstract

**Background:**

The provision of psychotherapy over distance using technology is a growing market reaching many patients and therefore the risks and benefits need to be known by all psychotherapists whether they themselves practice online or not. This comprehensive review of the main ethical arguments for and against different forms of online psychotherapy aims to enhance discussion of ethical issues in this growing area.

**Methods:**

A search of three databases (PubMed, PsycINFO, Web of Science) was conducted in August 2019 using a specific search protocol yielding 249 publications.

**Results:**

Of 24 ethical arguments in favor of online psychotherapy and 32 against, the top five ethical arguments in favor of online psychotherapy were (1) increased access to psychotherapy and service availability and flexibility; (2) therapy benefits and enhanced communication; (3) advantages related to specific client characteristics (e.g. remote location); (4) convenience, satisfaction, acceptance, and increased demand; and (5) economic advantages. The top five ethical arguments against engagement in online psychotherapy were (1) privacy, confidentiality, and security issues; (2) therapist competence and need for special training; (3) communication issues specific to technology; (4) research gaps; and (5) emergency issues.

**Conclusions:**

The findings may be of help to practitioners in deciding whether to engage in online psychotherapy, and in informing patients about risks and benefits, improving ethical guidelines, and stimulating further ethical discussion. The findings are argumentative and qualitative in nature, and further quantitative research is needed.

## Introduction

Technological innovation has led to rapid change in many professions, bringing both benefits and challenges. Since the late 1990s, a growing body of research has addressed issues related to online psychotherapy. To begin, that research focused mainly on the usefulness of online psychotherapy before shifting in emphasis to situations in which online psychotherapy might be used, with a view to evaluating the strengths and shortcomings of this approach (1).

The Joint Task Force for the Development of Telepsychology Guidelines for Psychologists (2) define telepsychology as “[…] the provision of psychological services using telecommunication technologies […]” (p. 792), which may be synchronous (real-time) or asynchronous, including “[…] telephone, mobile devices, interactive videoconferencing, e-mail, chat, text, and Internet […]” (p. 792). The service can be standalone or an adjunct to traditional psychotherapy. The various terms used to describe such services generally refer to psychotherapy delivered remotely using some form of communication technology. In this review, the term *online psychotherapy* encompasses all such terminological variants, including *telepsychology* [see (2)], *telepsychiatry* [see (1)], *online counseling* [see (3)], *behavioral telehealth*, *telemental health* [see (4)], *internet therapy* [see (5)], *internet counseling*, *online practice*, *online therapy*, *e-therapy*, *cyber-counseling*, *cyberpsychology*, *e-social work* [see (6)], and *e-mental health* [see (7)]. These terms may differ slightly according to professional context or technological modality.

The practice of online psychotherapy reflects the rapid evolution of technology and may include the use of “virtual reality, augmented reality, intelligent wearable devices, and artificial intelligence applications” [(8), p. 125]. As these technologies are still in the early stages of development, they are not included in this review. The present review includes online psychotherapy conducted by psychiatrists, psychologist, and social workers. Nurses are not included because of differences in their education and legal restrictions on the scope of their work.

As a growing number of patients seek online psychotherapy, therapists must consider the related ethical issues (3) and should be able to participate in this ethical discussion (9). For example, one important feature of a thorough process of informed consent is knowledge of risks and benefits in making a fully informed decision (10). One of the main barriers to further implementation of online psychotherapy is the uncertainty around ethical and legal issues (1), which are increasingly addressed in ethical codes of conduct (11).

The aim of this review is to summarize the main ethical arguments for and against the provision of online psychotherapy to further ethical discussion within the relevant professions, and to facilitate the development of comprehensive ethical guidelines to underpin the practice of online psychotherapy. This in turn will help practitioners in deciding to offer this form of psychotherapy and in helping their patients to make a truly informed decision about engaging in online psychotherapy. The present review is not about the quantity or volume of publications in which specific ethical arguments are discussed; rather, our concern was to identify the main ethical arguments to date as a sound basis for future discussion.

## Methods

Publications were collected in August 2019 from three databases: PubMed, PsycINFO, and Web of Science. These were selected on the basis that the topic relates to medical, psychological, and ethical issues.

To construct the search protocol, the research question was structured in terms of three topics: *ethics*, *psychotherapy*, and *online*. Synonyms and main terms for these three fields were selected, and a search code was constructed for each database (including MeSH terms for PubMed and Thesaurus of Psychological Index Terms for PsycINFO); for details, see *Full Search Code* in **Supplemental Material**). The search code was validated by an expert librarian at the University of Zurich.

Publication titles and abstracts were screened according to defined selection criteria (for details, see *Publication Selection Criteria* in **Supplemental Material**).

The main ethical arguments were extracted from the selected publications by JS, clustering text passages according to main topics. JM read the selected publications independently and clustered the publications under these main categories (**Table 1**). Disagreements were discussed and resolved.

**Table 1:** Ethical arguments in favor and against online psychotherapy.

Ethical arguments in favor of online psychotherapy	
Increased access, availability and flexibility	1–6, 8–23, 25–61, 63–70, 72–81, 83, 84, 86–105, 107–112, 114–229
Therapy benefits and enhancements in communication	1–43, 45–49, 51–58, 60, 61, 63–74, 76–79, 81–98, 100–102, 104–109, 111–115, 117, 119–124, 126, 128–158, 160, 161, 163, 165–167, 169–179, 181–188, 190–193, 196, 197, 199–217, 219–237
Client characteristics	1–4, 10–25, 27–37, 39–54, 56–61, 64, 66–68, 71, 72, 74, 76–84, 86–107, 110–121, 123, 125–131, 133–140, 142–148, 151, 152, 154–159, 161, 163, 168–172, 175–188, 190–198, 200–205, 207–209, 211–213, 215–217, 219, 222–224, 226, 228, 238
Convenience, satisfaction, acceptance and increased demand	1–3, 5, 8, 10–19, 21–35, 37–46, 48, 50–53, 55–57, 63–70, 72–74, 76–78, 80, 81, 83, 84, 86, 88–92, 94–96, 100, 101, 104, 106–108, 111–113, 115, 118, 120–122, 128–130, 132, 134, 135, 137, 139–141, 143, 144, 146–148, 152, 153, 155, 156, 159, 160, 163, 165, 169, 170, 172, 175, 176, 178–180, 184–188, 190–194, 197, 200, 201, 203, 206, 207, 209–213, 215, 217, 219, 223, 224, 226, 231, 235, 236, 238
Economic advantages	1, 3–5, 8, 10–16, 18, 20–24, 27–37, 39, 40, 42, 43, 45, 46, 49, 51–53, 56–58, 64–68, 73–78, 81, 83–88, 90, 91, 93, 96, 101–104, 107, 108, 110–112, 116–124, 126, 128–132, 134–136, 139, 140, 142–152, 154, 161, 162, 164, 165, 169, 170, 173, 175–179, 182–185, 187, 189–193, 197, 200–202, 207, 208, 210, 212–214, 216, 218–224, 226, 228–231, 238
Anonymity and privacy	3, 10, 11, 13–16, 18–23, 25, 27, 29–31, 33, 35, 37, 38, 41, 42, 47, 51, 56–58, 61, 64–67, 72, 76–79, 84, 86, 87, 89, 91, 95, 96, 98–101, 103, 105–107, 112, 115, 117, 120, 121, 128, 130, 133–135, 137, 139–141, 144, 146, 148, 149, 152, 155, 156, 158, 159, 163, 169–171, 176, 178–187, 189, 192, 198, 200, 201, 204, 207, 211, 212, 216, 217
Eliminating barriers to engage in psychotherapy	3, 5, 10, 12–16, 19–23, 25, 27, 29–33, 35–37, 41–43, 47, 51, 56, 58, 59, 61, 64, 66, 67, 69, 76, 77, 84, 86, 87, 89, 91, 92, 95, 96, 101, 102, 107, 109, 111–113, 117, 121, 128, 130, 132, 134, 135, 139, 140, 154, 156, 158, 159, 161, 163, 169, 170, 173, 176, 179, 181, 184, 185, 189, 194, 195, 197, 204, 213, 216, 217, 219, 224, 238
Therapeutic relationship	1, 3, 10–12, 14–16, 19, 20, 22, 23, 29–31, 35, 37–40, 43, 47, 49, 54, 56, 60, 61, 64, 66, 68–70, 72, 74, 78, 84, 85, 91, 94, 102, 107, 113, 117, 119, 121, 122, 124, 128, 133, 137, 139–141, 143, 144, 146, 147, 152, 154, 156, 161, 171, 172, 175–179, 181, 184, 186, 189, 197, 204, 209, 211–213, 215–217, 219, 224
Online teaching and supervision	1, 8, 14, 19, 21, 22, 32, 34, 35, 38–40, 42, 43, 45, 47, 48, 51–55, 60, 66–68, 74, 78, 83, 87, 88, 91, 94, 98, 99, 103, 104, 117–119, 130, 131, 134, 147–149, 156, 166, 169, 171, 172, 175–178, 181, 184–187, 191, 193, 200, 203, 204, 207, 211–213, 215, 222, 227, 229, 230, 234, 236, 238
Reducing stigma	3, 5, 8, 10, 11, 13–16, 19–23, 29, 31–33, 35, 36, 41, 42, 45, 51, 56, 64, 76, 78, 84, 86, 89, 91, 92, 95, 96, 101, 107, 109, 113, 133–136, 139, 140, 155, 156, 163, 171, 178–180, 182, 184, 191, 194, 204, 211, 216, 220, 224
Patient empowerment and increased patient control	1, 3–5, 7, 16, 19, 20, 30, 39, 42, 43, 49, 51, 52, 58, 60, 61, 69, 72, 74, 76, 78, 79, 81, 83, 86, 88, 90–92, 100, 104, 107, 113, 128, 132, 147, 156, 157, 169, 171, 177, 178, 184, 185, 187, 193, 196, 200, 204, 207, 208, 211, 216, 218, 219, 224, 238
Worldwide and cross-border psychotherapy	7, 12, 13, 19–21, 23, 25–27, 31, 32, 35–37, 45, 48, 50, 51, 57, 58, 61, 70, 76, 89–91, 99–103, 105, 113, 117, 118, 120, 127, 136, 138, 142, 146, 159, 163, 165, 169–171, 175, 176, 178, 181, 185, 200, 202–204, 211, 219
Emergencies	1, 3, 5, 12, 15, 19, 23, 25, 30, 33, 36, 37, 39, 42, 48, 49, 54, 57, 58, 61, 64, 69, 76, 80, 87, 91, 92, 97, 103, 104, 111, 113, 117, 126, 128, 132, 134, 139, 143, 148, 159, 168–170, 185, 193, 196, 207, 211, 212, 215, 216, 226
Adaptability of services and personalized care	1, 5, 7, 19, 21, 22, 30, 35, 39, 44, 45, 57, 86, 107, 108, 132, 135, 142, 152, 169, 175, 219, 224, 227, 228
Adherence and compliance	1, 8, 11, 13, 19, 20, 31, 46, 56, 72, 82, 88, 90, 93, 94, 114, 122, 132, 166, 175–177, 216, 219, 224
Opportunities for research	7, 13, 32, 43, 47, 52, 58, 61, 66, 67, 79, 83, 87, 108, 120, 121, 146, 185, 200, 203
Unethical not to provide online psychotherapy	29, 48, 52, 67, 103, 142, 155, 158, 159, 205–207, 216, 231
Freedom for therapists	17, 29, 49, 50, 67, 96, 107, 169, 187, 197, 200, 216
Enhancing accountability	42, 51, 52, 55, 150, 184–186, 204, 207
Protection of the therapist	23, 31, 53, 97, 143, 196, 207, 217
Social media	7, 26, 40, 54, 103, 155
Diminishing intimacy	12, 55, 67, 217
Informed consent	56, 57, 72, 117
Prohibition against free market	58
Ethical arguments against online psychotherapy	
Privacy, confidentiality and security issues	1–3, 5–20, 22, 23, 25–39, 41–74, 76–82, 84, 85, 87–105, 107, 108, 110–114, 116, 117, 119–122, 124, 127–134, 137–144, 146–150, 152–155, 157–181, 184–195, 197–201, 203–214, 216–219, 221–223, 225–236, 239–248
Therapists' competence and training	1–3, 5, 6, 8–12, 14–16, 18–27, 29–33, 35–39, 41–53, 55–58, 60–74, 76–78, 81, 82, 84, 85, 87, 89–96, 100–105, 107–110, 112–114, 116–122, 124–134, 137–144, 146, 147, 149, 150, 152–167, 169, 170, 172, 174–181, 184, 186–191, 193–197, 200, 203–206, 210–213, 218, 219, 221, 223–229, 232–235, 239–242, 244, 246–248
Communication issues	2, 3, 5, 7, 9–17, 19, 20, 22, 23, 25–35, 37, 39, 41–48, 51, 52, 55–61, 63–70, 72–74, 76–81, 84–95, 97–103, 105, 107, 111–113, 115–120, 124, 126–132, 134, 136–144, 146–150, 152–159, 164–181, 183–189, 191–193, 195–197, 200, 201, 203, 204, 206–209, 211–213, 216, 217, 219, 223–227, 230, 232–236, 239, 241–244, 247–249
Research gaps	1–3, 5–7, 9, 11, 13–19, 22–24, 27–35, 37–39, 41–43, 45–47, 49, 51–61, 64–74, 76–80, 82, 84–92, 96, 98, 100, 103, 104, 107, 111–114, 117–119, 121–124, 127, 128, 130–132, 134, 137, 140–144, 146, 147, 149, 151, 152, 154–158, 164, 169–173, 175–178, 180, 181, 183–185, 187, 189, 191–197, 201, 203, 204, 207, 211–220, 224, 227, 228, 231, 233, 235, 237–239, 241, 244, 245, 249
Emergency issues	2, 3, 5, 9–13, 15–19, 22, 23, 25, 27, 28, 30–32, 34–37, 41–43, 45, 48, 50–61, 63–77, 79, 80, 84, 88, 89, 91–95, 97, 98, 101–105, 111, 113, 114, 116–120, 124, 126–129, 132–134, 137–140, 143, 144, 146, 148–150, 152–156, 158, 159, 162, 164, 165, 167–169, 174–176, 178–181, 185–187, 189, 191, 192, 195–199, 201, 203, 204, 206–209, 211, 212, 216–219, 223, 225–227, 234, 235, 241–245, 247
Informed consent issues	1, 2, 6, 9–12, 14, 16, 18, 19, 22, 23, 25, 27–30, 32, 35–37, 41, 43–46, 48, 49, 51–58, 60, 63–67, 69–74, 77, 78, 81, 84, 88, 89, 91, 94, 95, 102–105, 113, 114, 116–121, 124, 127, 129–134, 138–142, 144, 146, 148–150, 153, 155, 158–161, 163–167, 169, 171, 173–175, 178–181, 183, 184, 186, 187, 189, 191, 192, 194, 195, 197, 199, 201, 203, 204, 206–212, 218, 219, 221, 223–228, 231, 233–235, 241–244, 247, 248
Technological competence	1–3, 5, 9, 11, 12, 14, 15, 19, 20, 22–25, 27–32, 35, 37, 41, 43–45, 48–53, 56, 57, 59–71, 73, 74, 78, 80, 81, 87, 89–95, 101, 104, 105, 107–110, 112–114, 116–122, 124, 128–130, 132, 134, 138–142, 144, 146, 147, 150, 152, 153, 155, 156, 159–161, 163, 164, 166, 167, 169, 174–182, 184, 188, 189, 191–193, 197, 200, 201, 203–206, 211–214, 219, 223, 225–227, 229, 230, 232, 234, 239–241, 245–247
Absent or incomplete guidelines	1, 3, 4, 7, 9, 10, 12, 14–16, 18, 20, 22–24, 28–32, 34–37, 39, 42, 43, 45–50, 52, 53, 55, 57, 59–61, 63–65, 67, 68, 70–75, 77–81, 84–87, 89–91, 93, 94, 96, 97, 100, 102–105, 107, 110, 112, 113, 116, 118, 120–122, 129–131, 134, 136, 137, 140–143, 146–150, 152, 154, 155, 157–159, 161, 165, 169–173, 176, 177, 180, 181, 185, 186, 189, 191, 192, 194–196, 198, 203–205, 207, 212, 213, 216, 217, 223, 224, 226–228, 231, 233, 235, 237, 239, 241, 243–245, 247
Legal issues	1–4, 6, 9, 10, 12, 13, 15, 16, 18, 19, 23–27, 29–32, 35–37, 41–45, 49–51, 53, 55–59, 61, 64–68, 70, 74–77, 79, 84, 85, 87, 89–94, 96, 97, 102, 105, 107, 108, 111–114, 116–119, 121, 124, 126, 128–131, 134, 136, 137, 140–144, 146, 148–150, 154, 155, 158, 159, 161, 164, 167, 169–171, 173, 176–180, 183, 185, 186, 189, 191, 195, 196, 200, 201, 203, 207, 210–212, 214, 216–219, 221, 223–225, 227, 229, 231, 237, 239, 243–245, 247
Practicing across borders	2, 3, 6, 9, 10, 12–20, 22, 23, 25–27, 29–32, 35–37, 41–43, 45, 48–51, 53, 55–59, 61, 64–77, 79, 85, 87, 89–92, 94, 96, 97, 99, 100, 102–105, 109, 113, 114, 116–120, 128–130, 133, 134, 136, 137, 140, 142, 143, 146, 148, 149, 154, 155, 158, 159, 163, 164, 168–171, 173, 174, 176, 180, 181, 183, 185, 186, 189, 191, 195–198, 200, 201, 203, 204, 210–212, 218, 221, 223, 225–227, 229–231, 239, 243–247
Patient characteristics	2, 3, 10–13, 15–20, 22, 24, 26, 27, 30–34, 37, 39, 41–45, 48, 51–53, 58, 59, 61, 63, 64, 66–74, 76–78, 80, 81, 84, 87–89, 91, 92, 95, 97, 103–105, 107, 112–114, 117–122, 128, 131–133, 137, 139, 140, 142, 144, 146, 148, 150, 152, 153, 155, 156, 158, 159, 163, 168, 169, 174, 176, 177, 179–181, 184, 185, 191–193, 195, 196, 198, 204, 208, 209, 211, 212, 214, 216, 217, 219, 223–226, 235, 236, 238, 239, 248
Technical issues	1–3, 10, 11, 13, 16–20, 22, 23, 26, 29–32, 34, 36, 37, 39, 41, 45–48, 51, 52, 56, 57, 59, 62–67, 69, 71, 73, 76–79, 84, 89–91, 94, 95, 101, 103–105, 111, 113, 114, 116, 117, 119, 120, 127–129, 131–134, 137, 139–142, 144, 147–150, 152, 153, 155, 156, 158, 162–164, 166–169, 171, 174–176, 178, 180, 181, 183, 184, 186, 189–192, 197, 200, 203, 204, 209–213, 217, 219, 223, 225, 226, 232, 235, 236, 239, 243, 244
Payment and insurance issues	2, 3, 6–10, 12, 15–17, 19, 20, 22, 23, 25, 26, 29–32, 35–37, 41–43, 45, 48, 53, 55, 58, 59, 64–71, 73–77, 84, 88–91, 94, 95, 99, 100, 105, 113, 114, 116–119, 124, 128, 129, 133, 134, 137, 138, 140–142, 146, 149, 150, 154, 155, 158, 159, 162, 164, 166, 167, 169, 171, 173–177, 179, 180, 188, 191–194, 197, 201, 203, 207, 208, 210, 211, 216–219, 221, 223–225, 230, 239, 241, 243–245, 247
Therapeutic relationship issues	1, 3–5, 7, 11–13, 15, 16, 19, 20, 22, 23, 26–28, 30–32, 34, 35, 37, 39, 43–49, 51, 52, 54–56, 58–61, 63–68, 72, 75, 78–81, 84–87, 90, 91, 93, 94, 100, 102–104, 107, 111, 113, 118, 120, 126, 128, 130, 132–134, 136, 139, 141–143, 146, 147, 149, 153, 155, 156, 158, 159, 169, 171, 172, 174, 175, 181, 184–186, 188, 189, 191, 195, 197, 199, 201, 203, 204, 207–209, 211–213, 217, 230, 233, 239, 245, 248
Availability and access issues	1, 5, 7, 9, 10, 12, 15, 18–20, 22, 23, 26, 29, 30, 32, 35, 37–39, 41, 42, 44, 45, 47, 48, 51, 54–58, 60, 61, 64, 65, 67, 69, 73, 74, 77–81, 84, 87–89, 91–93, 95, 101, 103–105, 113, 114, 117–119, 121, 124, 126, 129, 130, 132, 134, 144, 149, 150, 152, 153, 155, 156, 158, 160, 163, 164, 167, 169, 172, 174, 177, 180, 181, 184–189, 191, 193, 195, 199–201, 203, 204, 206, 207, 211–213, 223, 225–227, 234, 235, 238, 239, 241, 242
Identity and verification issues	3, 5, 9, 11–13, 16, 19, 22, 23, 25, 27, 29, 30, 32, 35, 37, 41–43, 45, 47, 51, 55–59, 63–73, 75–77, 79, 84, 89, 91, 92, 95, 96, 98, 100–105, 107, 112, 113, 116, 117, 119–121, 126–129, 134, 137–140, 144, 146, 148–150, 154–156, 158, 159, 162, 164, 169, 171, 172, 177, 179–181, 183, 186, 191, 192, 194, 195, 197–199, 201, 203, 204, 206, 210, 211, 223, 225, 226, 230, 234, 235, 241, 242, 245
Image, tradition and therapists' attitude	1, 5, 13, 15–17, 19, 24, 28, 29, 32, 37, 39, 43, 47, 50, 52, 54, 58–60, 66–68, 72–74, 78–80, 82, 84, 89, 91–93, 96–98, 104, 107, 108, 110, 112, 113, 124, 131, 134, 137–139, 142–144, 147, 148, 150, 155, 156, 158, 159, 172, 180, 184–187, 191, 195, 197, 200, 203, 207, 211, 216, 217, 219, 224, 226, 227, 233, 243–245, 249
Misuse and harm	12, 15, 18, 19, 23, 29, 30, 33, 37, 41–43, 47, 51, 55, 56, 58, 59, 61, 64, 70, 72, 74, 78, 81, 84, 85, 87, 89, 94, 95, 101–103, 105, 107, 117, 119, 120, 124, 133, 136, 138, 143, 146, 148–150, 156, 177, 180, 181, 184–186, 195, 200, 203, 223, 239, 244
Boundary issues	2, 3, 10, 16, 19, 26, 31, 38, 39, 48, 51, 54, 56–58, 60, 63, 67, 71, 73, 74, 80, 82, 88, 89, 93, 95, 103, 113, 120, 124, 126, 130, 133, 134, 136, 143, 155, 156, 160, 172, 174, 177–179, 188, 189, 191, 192, 206, 211, 223, 225–228, 236, 239, 242
Comparability to in-person treatment	1–3, 7, 14, 16, 19, 29, 30, 32, 33, 39, 42–44, 46, 56, 57, 64, 66, 67, 70, 72, 76, 80, 81, 84, 86, 90, 91, 104, 111–114, 117–119, 126, 134, 136, 155, 159, 179, 191, 192, 195, 198, 201, 203, 204, 221, 226, 233, 244, 245, 247, 249
Increased costs	1, 3, 9, 12, 16, 21, 23, 32–34, 36, 39, 42, 45, 48, 49, 51–53, 58, 61, 64, 65, 68, 83, 87, 89, 91, 97, 99, 101, 128, 129, 132, 143, 144, 147, 154, 159, 162, 169, 171, 180, 201–204, 207, 213, 214, 219, 223, 229, 231, 239, 248
Increased liability and litigation	3, 15, 22, 23, 29, 32, 35–37, 42, 52, 57, 59, 67, 68, 70, 74, 77, 80, 89, 92, 94, 96, 97, 113, 124, 126, 129, 130, 134, 142–144, 158, 164, 165, 169, 180, 181, 183, 189, 194–198, 201, 207, 218, 231, 233, 243, 245
Negative influence of technology use	5, 7, 10, 20, 47, 54–56, 60, 61, 64, 66, 67, 69, 72, 84–86, 95, 98, 108, 109, 115, 121, 128, 133, 134, 137, 140, 152, 157, 158, 166, 169, 172, 179, 181, 203, 211, 216, 217, 223, 237, 249
Social media	2, 3, 10, 18, 26, 48, 54, 57, 60, 63, 69, 71, 93, 103, 105, 113, 120, 130, 134, 155, 157, 160, 164, 172, 174, 188, 189, 205, 206, 211, 223, 226, 227, 242, 247
Financial gain	9, 23, 25, 27, 30, 44, 45, 49, 58, 59, 72, 76, 81, 85, 90, 92, 100, 102, 103, 117, 120, 126, 130, 144, 164, 169, 180, 186, 189, 208, 224, 226, 241
Loss of therapeutic control	16, 17, 26, 28, 41, 45, 48, 57, 60, 63, 73, 80, 86, 124, 126, 130, 134, 138, 164, 166, 181, 196, 207, 208, 211, 217, 247
Adherence issues	10, 11, 16, 20, 41, 69, 75, 87, 101, 103, 132, 152, 154, 156, 165, 169, 187, 191, 196, 208, 211
Online supervision and teaching issues	2, 19, 54, 66, 116, 126, 157, 159, 186, 226, 236, 239
Dependence and loss of control by the patient	48, 58, 88, 107, 177, 206, 207, 216, 219
Autonomy issues	28, 45, 48, 60, 81, 132, 134
Dehumanization	15, 28, 68, 89, 90, 178
Stigmatization	29, 91, 113, 138

The publications are depicted in numbers (for full citation, see *References*).

## Results

The collection of publications in August 2019 in the three databases PubMed, PsycINFO, and Web of Science and selection according to the selection criteria resulted in a final sample of 249 publications.

The final sample (n = 249) included 179 articles, 6 books, 55 book chapters, 2 doctoral theses, 5 book reviews, 1 brief communication, and 1 item of correspondence. Among these, there were 32 literature reviews, 2 systematic reviews (regarding the socioeconomic impact of telehealth and guidelines in videoconferencing), 6 editorials or introductions, 5 book reviews, and 6 books (including 4 practice guides). Additionally, there was one case study, one correspondence, one research digest, one discussion, and one paper on ethical reasoning. In total, 30 of the selected publications were empirical studies, including 14 surveys and 6 website analyses. Six related to specific mental illnesses, one was a comparison of in-person and online psychotherapy, two included practitioner interviews, one was a study protocol for a meta-analysis, and one related to a practitioner discussion forum. Among other notable features of the final sample, 18 included guidelines, and 25 focused on specific countries.

The selected publications ranged across disciplines that included psychology (70), psychiatry (33), psychology and psychiatry (2), social work (12), and telemedicine (6). The remaining publications (125) related to psychotherapy in general, with no specific disciplinary focus. In terms of technological modality, these included email (13), telephone (5), videoconferencing (28), text-based methods including email and chat (14), email and text messages via cellphone (3), mobile phones (3), text messages (1), email and social media (2), and videoconferencing and mobile phones (6). The remaining 173 related to communication technologies in general rather than any specific technology or mode of communication.

**Table 1** summarizes the main ethical arguments (24 pro and 32 contra) extracted from the final 249 publications, organized by number of mentions. The next section describes the various categories. For clarity, the summary does not include all discussion points, and reference is made to only one publication for each ethical argument. For further information about the arguments, readers are referred to the source (**Table 1**).

### Ethical Arguments in Favor of Online Psychotherapy

#### Increased Access, Availability, and Flexibility

Online psychotherapy can improve and enhance access to health care services and evidence-based care, especially for those living in rural or remote areas and populations that are underserved for other reasons [see (12)]. Services can be accessed anywhere and at any time, allowing greater flexibility [see (13)]. This is advantageous for both therapist and patient, enabling immediate and timely care [see (3)]. Online psychotherapy may also facilitate more frequent contact between patient and therapist [see (14)], and as more therapists are available to the patient, specialist care is easier to access, and the range of services is wider [see (1)].

#### Therapy Benefits and Enhancements in Communication

According to a growing number of favorable research findings, online psychotherapy can be as efficient, effective, and efficacious as traditional therapy (or more so) [see (1)]. Multiple therapeutic orientations and modalities are translated into online communication, but cognitive behavioral approaches seem to be most appropriate or the easiest to transfer [see (15)]. For example, during an *in vivo* exposure, the therapist could be virtually present [see (16)]. However, also other psychotherapeutic orientations such as psychoanalysis assess and discuss ethical issues of practicing online [see (17)]. Online psychotherapy offers a viable alternative to in-person treatment but can also be used as a supplement or adjunct [see (18)]. This affords new opportunities for creative approaches involving different models of therapy and technological modalities, and additional online material (websites, videos, etc.) are easily integrated into therapy [see (19)]. Data recording and documentation of the online therapeutic process is also easier, allowing treatment, treatment stages, and therapeutic techniques used by therapist and patient to be revisited [see (20)].

#### Client Characteristics

Online psychotherapy can be especially useful for clients living in geographically remote, rural, or otherwise underserved areas where few or no therapists are available [see (21)], as well as for homebound or mobility-impaired patients [see (22)]. Access to traditional in-person services may be limited by the psychiatric condition itself, as in cases of agoraphobia, anxiety, or other illnesses that restrict physical encounter, and online psychotherapy again offers a possible solution [see (23)]. Online psychotherapy seems especially appropriate for patients with mild or moderate symptoms [see (24)], but might also be a viable tool for patient in acute crisis with no possibility for immediate in-person care [see (25)].

#### Convenience, Satisfaction, Acceptance, and Increased Demand

Online psychotherapy is perceived as convenient and comfortable by patients and therapists alike [see (26)], not least because of the greater flexibility it offers in terms of location and time [see (27)]. Online services have gained increasing acceptance among patients and therapists [see (28)], who express satisfaction with this approach [see (15)]. Unsurprisingly, then, demand and interest is on the increase among both patients and practitioners [see (29)].

#### Economic Advantages

Online psychotherapy is reported to be more cost-efficient [see (30)], with the potential to reduce healthcare costs for patients, therapists, and society as a whole [see (31)]. As a single therapist can reach more patients, especially in underserved populations [see (32)], long waiting lists for face-to-face treatment can be reduced [see (33)], offering a possible solution to the workforce shortage in mental health provision [see (34)]. Online psychotherapy might pose a solution to an undersupply in mental health care in various regions of the world, especially in low- and middle income or developing countries, for example, in India [see (34)].

#### Anonymity and Privacy

Because online psychotherapy can be provided anonymously and one is not seen entering the therapist's office [see (20)], it can enhance the patient's sense of anonymity and privacy [see (13)]. Perceived or actual anonymity may lead in turn to reduced inhibition and greater openness in discussing emotional topics [see (35)].

#### Eliminating Barriers to Engagement

By reducing or eliminating barriers such as fear of social stigma, online psychotherapy can reach patients who might never have sought traditional in-person therapy [see (36)]. This might serve as an entry point to the mental health system, including traditional in-person therapy as a possible next step [see (37)].

#### Therapeutic Relationship

The therapeutic relationship established in online psychotherapy is commonly perceived as equal to or better than in-person therapy, and an established therapeutic relationship can be enhanced using online communication [see (38)].

#### Online Teaching and Supervision

Technology-mediated communication can contribute positively to teaching and supervision and facilitates inter-professional and inter-collegial exchange worldwide [see (39)]. Online psychotherapy conducted by email or other text-based communication automatically generates a record of the sessions [see (32)], and a videoconferencing approach enables sessions to be videotaped [see (40)] for later supervision.

#### Reducing Stigma

Any stigma, stigmatization, or perceived stigma associated with seeking mental help services or concerns about being stereotyped can be reduced or eliminated by online psychotherapy. This may in turn help to address barriers to traditional psychotherapy such as concerns about anonymity and privacy [see (41)].

#### Patient Empowerment and Increased Patient Control

Online psychotherapy empowers the patient because, for example, it is much easier to move to another therapist [see (42)], giving the patient more control over their therapy [see (43)]. This reconfigures the balance of power between therapist and patient, making the interaction more collaborative [see (20)].

#### Worldwide and Cross-Border Psychotherapy

Online psychotherapy can be provided from anywhere, without regard to geographical boundaries, state lines, national borders, or time-zones, allowing therapists to reach patients who are, for instance, temporarily abroad [see (19)].

#### Emergencies

Online psychotherapy can be useful for emergencies and crisis interventions. As compared to traditional in-person therapy, it may provide more immediate access to services, and disclosure of suicidal or homicidal tendencies may be easier online [see (19)]. In the context of crisis and suicide prevention, suicide hotlines and other forms of telephone emergency care are long established and proven practices [see (37)].

#### Adaptability of Services and Personalized Care

Online psychotherapy can offer services that specifically match patients' needs [see (19)], facilitating genuinely patient-centered care [see (44)] and individualized treatment and technology options [see (45)].

#### Adherence and Compliance

Levels of adherence, attendance, and compliance as good as or better than in-person treatment can be achieved using online psychotherapy [see (46)].

#### Opportunities for Research

Online psychotherapy offers unique opportunities for research [see (32)]; for example, email-based therapy automatically creates a written record, which can be used for research purposes [see (47)].

#### Unethical Not to Provide Online Psychotherapy

Failure to provide online psychotherapy to vulnerable people who need it can be seen as unethical—for example, patients living in rural or remote areas with few or occasional local options [see (48)].

#### Freedom for Therapists

Online psychotherapy can afford the therapist greater freedom [see (49)], including more professional opportunities and a better balance between professional and private life [see (50)].

#### Enhancing Accountability

Online psychotherapy increases the accountability of both therapist and patient, not least because it is easier to keep records and to make transcripts available to both parties [see (51)], reducing the potential for malpractice and litigation [see (52)].

#### Protection of the Therapist

Security issues raised by risky environments or when communicating with potentially dangerous patients can be reduced by online service provision [see (53)].

#### Social Media

Offering unprecedented opportunities for access and connecting with patients and other therapists, social media can be a useful therapeutic tool [see (54)].

#### Diminishing Intimacy

As the distance provided by technology inhibits physical proximity, online psychotherapy can help to reduce the risk of patient-therapist (sexual) intimacy [see (55)].

#### Informed Consent

The informed consent process can be enhanced by online communication—for example, web pages can be revisited (56), with links to additional information resources or technical material and translation into different languages [see (57)].

#### Prohibition Against Free Market

The view that one should not engage in online psychotherapy is legally problematic because it restricts trade and the ethical right to a free market (58).

### Ethical Arguments Against Online Psychotherapy

#### Privacy, Confidentiality, and Security Issues

Among concerns about privacy, confidentiality, security, and safety in online psychotherapy [see (59)], one relates to the use of unsecured websites or unencrypted communication tools, like commercially available software [see (60)] that is easily hacked [see (61)]. Data security may also be compromised when technology fails [see (62)], with potential breaches of confidentiality that might extend beyond the therapist's control [see (63)].

#### Therapist Competence and Training

To provide online psychotherapy, training is needed to ensure appropriate technology-related competences, as well as clinical and therapeutic competences specific to the online setting. In particular, the therapist would require knowledge of ethical approaches and guidelines, as well as specific legal requirements and policies [see (18)]. In general, therapeutic skills in in-person contact do not automatically translate into online therapeutic skills [see (32)]. At present, standards are not well defined, and there are few training or education programs for online psychotherapy, which is not included in most traditional curricula [see (64)]. In relation to working remotely with patients in other countries, the therapist would need to be familiar with international laws and legal requirements in the patient's jurisdiction, and additional cultural competences might also be required [see (65)].

#### Communication Issues

Among negative issues, one of the most widely discussed is the absence of non-verbal cues in the therapeutic interaction, especially when using text-based media but also when using telephone or videoconferencing, which may lead to misunderstandings and miscommunication [see (20)]. If a therapist were to miss some important item of clinical information, the whole diagnostic process and psychological assessment could be impaired [see (28)]. The use of e-mail in this context can undermine the conversation in terms of time lag and lack of spontaneity, and it may prove difficult to express empathy, warmth, and feelings [see (64)]. For these reasons, online psychotherapy may not be appropriate for all therapeutic approaches and modalities [see (66)].

#### Research Gaps

Many authors claim that there is insufficient research in support of online psychotherapy or that there are too many knowledge gaps, especially with regard to effectiveness, efficacy, and long-term outcomes and as compared to in-person treatment [see (30)].

#### Emergency Issues

Questions also arise as to whether an emergency or crisis situation involving threat to self or others can be detected and addressed where patient and therapist are at different locations [see (67)]. Other ethical issues regarding emergency or crisis situations include verification of patient identity and location [see (68)], technological difficulties [see (69)], and cross-border practice [see (70)].

#### Informed Consent Issues

In light of the many differences from in-person therapy (e.g. technical, legal), online psychotherapy requires a particular form of informed consent [see (71)]. However, it might be difficult to determine whether the patient is legally able to give consent or to assess their mental capacity to do so [see (72)].

#### Technological Competence

A therapist's lack of technological competence and patient and therapist awareness of their respective skills are important issues in this context, as discomfort or fear of using technology is not uncommon [see (73)].

#### Absent or Incomplete Guidelines

Regulatory guidelines and standards of practice or care in this area are considered incomplete or absent. Guidance by legal or regulatory bodies is also lacking, especially in terms of global or international regulation of cross-border practice, and the absence of specific ethical guidelines or codes of conduct for online psychotherapy leaves many ethical questions unanswered [see (42)].

#### Legal Issues

Unresolved jurisdiction and few or no specific laws governing licensing, certification, training and education, informed consent, and cross-border practice are problematic issues for online psychotherapy [see (74)].

#### Practicing Across Borders

Many issues arise in relation to online psychotherapy conducted across state or national borders, including legislative, licensing, and cultural differences [see (30)]. For instance, it may be unclear whether the therapy is seen to take place at the patient's or the therapist's location, raising such questions as which jurisdiction is responsible when a problem arises or which regulates professional practice in the event of a violation [see (75)]. The therapist may not even know or cannot be sure where the patient is located, especially if they choose to remain anonymous [see (76)]. Cultural differences between patient and therapist might influence the communication itself by different cultural behaviors or language use resulting in different interpretations of the behavior and potential misunderstandings [see (30)].

#### Patient Characteristics

Online psychotherapy may not be suitable for all patients, clinical conditions, psychiatric disorders, and problems; it may sometimes be contraindicated, especially in the case of severe mental disorder, or for patients who are highly dysfunctional and/or pose a threat to themselves or others [see (16)]. A patient's inability, diminished competence, or discomfort when using technology might also be considered a barrier [see (15)].

#### Technical Issues

Technical difficulties and failures are major concerns in this context, possibly leading to frustration and anger, which may be distracting or disturbing [see (73)].

#### Payment and Insurance Issues

Payment, reimbursement, fee structure, and billing for online psychotherapy raise many questions, such as how interruption or technical failure will be handled [see (77)]. Another important issue is whether insurers will cover online psychotherapy in general, as well as instances of malpractice or liability, which may become especially complicated across state or national borders [see (68)].

#### Therapeutic Relationship Issues

Many authors have questioned whether an effective and successful therapeutic alliance can be developed solely through technology [see (78)] and whether the well-known benefits of the therapeutic relationship might disappear or diminish in online psychotherapy [see (79)]. Other issues raised in this include absence of non-verbal cues and lack of intimacy [see (51)].

#### Availability and Access Issues

Because technology often creates a sense of permanent access, this may become a problem, as the therapist cannot and will not guarantee this [see (60)]. Response time and delays are also an important issue, especially in emergency situations [see (80)]. Additionally, accessibility (for instance, in terms of technology, devices, connectivity, and applications) may be restricted for people of lower socioeconomic status or for those unable to use the equipment [see (32)].

#### Identity and Verification Issues

As it may be difficult to verify the identity of the patient (or the therapist) online, deception or fraud is a possibility—for example, a therapist might inadvertently treat a minor without parental consent [see (64)].

#### Image, Tradition, and Therapist Attitude

Many therapists have a negative view of online psychotherapy and are clearly concerned or strictly against it, with poor reported satisfaction and acceptance among therapists [see (1)] and concerns that online psychotherapy might damage the profession's image [see (58)].

#### Misuse and Harm

Unethical, malign, or abusive behavior may be easier online [see (81)]—for instance, practicing without a license or without appropriate training, or even pretending to be a therapist [see (23)].

#### Boundary Issues

Online psychotherapy may make it more difficult to maintain professional boundaries, posing a threat to the professional relationship—for example, an interaction mediated by technology might seem social, conversational, or less formal, and the flexibility of location and time might lead to communication in inappropriate locations or at odd times, as the therapist might be tempted to communicate while on holidays, traveling, or while ill [see (26)]. As another potential threat to boundaries, therapists and/or patients might use search engines to explore private information [see (82)].

#### Comparability to In-Person Treatment

One important open question is whether online psychotherapy is truly comparable to in-person treatment, and whether it can replace traditional in-person therapy. Many authors have expressed doubts in this regard, to the extent that some believe online services may prevent patients from seeking more suitable traditional therapy [see (56)].

#### Costs

Online psychotherapy entails some initial costs for the therapist, which may make access to online psychotherapy services too expensive for some patients [see (68)]. These initial costs could make it difficult to implement online psychotherapy in some low-income and developing countries [see (83)].

#### Increased Liability and Litigation

Therapists who provide an online service may be more exposed to litigation and increased liability, as for example in cross-border cases [see (29)].

#### Negative Influence of Technology Use

Online psychotherapy may contribute to internet overuse and ultimately internet addiction [see (47)], potentially increasing social isolation [see (84)] and exposure to unregulated and misleading health-related or other information [see (85)].

#### Social Media

The use of social networking sites poses new ethical challenges and invites potentially unethical interactions in the context of online psychotherapy, such as friend requests from patients [see (57)] or problematic self-disclosure [see (18)].

#### Financial Gain

There is a danger that online psychotherapy might be conducted for financial gain without due regard to the best interests of the patient [see (27)].

#### Loss of Therapeutic Control

Online psychotherapy may risk loss of therapeutic control [see (86)]—for example, in relation to the patient's location [see (57)].

#### Adherence Issues

Compliance and adherence to therapy may be undermined in an online setting, given the ease of dropping out, logging off, hanging up the phone, or terminating the connection [see (87)].

#### Online Supervision and Teaching Issues

Supervising and teaching online raises a number of specific ethical issues and questions [see (66)].

#### Patient Dependence and Loss of Control

In online psychotherapy, the patient may experience less control (58), and the process may foster dependence [see (88)].

#### Autonomy Issues

Online psychotherapy may hamper patient autonomy [see (28)]—for example, a patient may experience a sense of intrusion when receiving online psychotherapy at home [see (81)].

#### Dehumanization

Online psychotherapy may lead to dehumanization of the therapeutic environment [see (89)] or of the patient if experienced as intrusive by someone who is already vulnerable [see (90)].

#### Stigmatization

An online setting may promote inadvertent discrimination or cultural insensitivity by masking important cues [see (91)].

## Discussion

Online psychotherapy offers many advantages like benefits for the therapeutic process and the therapeutic communication itself, also by being more convenient than traditional settings of psychotherapy. Both many patients and therapists seem to be satisfied with the use of online psychotherapy. Not surprisingly, this kind of psychotherapy is increasingly being used. Online psychotherapy promises to solve economic pressures by being more cost-effective, offering a solution for workforce shortage problems and increasing access to necessary psychotherapy for many different populations suffering from mental health problems who might be difficult to reach such as patients living in rural areas. Reducing barriers to engaging in psychotherapy by reducing stigma, being able to attend online sessions anonymously thus enhancing a sense of privacy, reaching patients worldwide and across borders are other advantages of online psychotherapy. Conducting psychotherapy online gives the possibility to adapt services to specific patients offering more personalized care, enhancing patient control, and empowering the patient resulting in more adherence to and compliance with the treatment itself. The specific setting of online psychotherapy gives the therapist more freedom and offers physical protection, but might also protect the patient from breaches of intimacy boundaries and enhance the accountability of both patient and therapist. Additionally, online psychotherapy offers new opportunities for research, teaching, and supervision, enhancing the informed consent process, offering new opportunities through the use of social media and might give good assistance in emergency situations. Online psychotherapy might even improve and extend the therapeutic relationship.

One of the biggest and most discussed disadvantages of using psychotherapy is the risks with regard to privacy, confidentiality, and data security. Online psychotherapy creates new challenges to therapist competences which brings about the need for new forms of training and education, especially technological competences regarding many technical issues that might occur. Technological competence is not only needed by the therapist, but also by the patient. New communication skills are needed and particular attention must be paid to the development of the therapeutic relationship regarding the many boundary issues that might occur. Difficulties herein are put up by new access and availability issues and the loss of therapeutic control. Broader research, new guidelines, and a consideration of legal issues in general are needed especially regarding the practice across borders of nations, new payment and insurance issues, challenges to the informed consent process, dealing with emergency situations, enhancing the identification process of the therapist and the patient, and selecting patients that are suitable for online psychotherapy. The comparability to in-person treatment might be questioned and some fear danger to the image of psychotherapy. Therapists might fear increased liability and litigation. Online, new forms of misuse are possible and charlatans might utilize this kind of psychotherapy to achieve financial gains. Other feared disadvantages of online psychotherapy are the dehumanization and stigmatization of patients, patient dependence, and loss of patients' autonomy. New adherence issues might occur regarding the ease of ending an online session. Online supervision and teaching and the use of social media raise further ethical questions. Extensive costs might be faced by patient and therapists, when using online psychotherapy, for example, to set up the new technologies. Last but not least, the negative influence of technology itself might endanger its users.

This review has a number of limitations. First, only articles in English and German were included. However, only fourteen of the articles that were found to meet the search criteria were in a different language and therefore excluded. Among the emerging research topics not included in this review are online training and supervision, social media, avatar, second life, robots and bots, artificial intelligence, computer-mediated (self-help) therapy, psychology-related smartphone apps, internet-based group therapy and telecare, online forums, open chat, therapy for older adults, therapy for children and adolescents, and marriage and family therapy.

It is beyond the scope of the present review to offer exhaustive recommendations for clinical practice or how these ethical risks might be resolved in practice, and further systematic research should more fully address this topic. For some recommendations directly deducted from the results of this review, see **Table 2**.

**Table 2:** Recommendations for practice.

Thorough protection of privacy of the patient, ensuring confidentiality, and security Engaging in special training and establishing special competence needed when conducting online psychotherapy, such as technological competences Being aware of communication challenges of the respective medium used, such as missing of non-verbal cues when using email Preparing for emergencies, for example, by establishing emergency plans, and being prepared to contact a local professional being able to intervene if necessary Being aware and reassure the true identity, age, and location of the patient Giving the patient the opportunity to reassure the true identity of the therapist and his/her certifications Set up an exhaustive informed consent form and thoroughly discuss all the risks and benefits with the patient in order to enable her/him to make a truly informed decision about engaging in online psychotherapy Clarifying fee and insurance issues Being aware of boundary issues with regard to the establishment and maintenance of a professional therapeutic relationship online Offering adequate anonymity and privacy to help eliminating barriers in engaging in psychotherapy Adapt services to the particular needs of the patient, thus offering personalized care whenever possible Be open toward further research on online psychotherapy, especially in cross-border online psychotherapy Support and welcome the establishment of new guidelines for conducting ethical online psychotherapy

Counting the frequency of arguments does not clarify their relative importance; to evaluate their true weight, a more quantitative survey of experts' ratings is needed. Without that deeper understanding, the risks and benefits reviewed here remain anecdotal and qualitative, with only limited validity (92).

In future systematic research on efficacy, effectiveness, and efficiency of online psychotherapy is needed and practice guidelines, legal and ethical frameworks need to be developed. Further research in the fast growing field of online psychotherapy seems vital. Some important topics requiring further investigation are summarized in **Table 3**.

**Table 3:** Recommendations for future research in online psychotherapy.

Systematic research on the efficacy, effectiveness, efficiency, and comparability of online psychotherapy to in-person psychotherapy regarding different technologies, different mental disorders, and severity of symptoms Translatability of different therapeutic orientations in online psychotherapy by assessing efficacy, effectiveness, efficiency, and comparability to the in-person setting, alterations needed, and suitability of different technologies for the respective therapeutic orientation Possibility and applicability of certain clinical practices like online prescription, diagnosis, assessment of suicidality or homicidality, assessing decision-making capacity for informed consent regarding the usage of different technologies Assessing which client characteristics are suitable for online psychotherapy and which are contraindicated, also regarding different technology use like video-conferencing or e-mail, as standalone or adjunct to in-person sessions, particular handling of homicidal or suicidal patients Research regarding cross-border, worldwide, and cross-cultural practice by assessing legal issues, influence of cultural factors, language and communication difficulties, patient-therapist fit, malpractice, payment and insurance issues, acquirement of special competences Assessing the changes in the therapeutic relationship due to different communication technologies used in online psychotherapy and the new forms of abuse that might appear or be possible in online psychotherapy compared to in-person psychotherapy Research on additional skills needed by psychotherapists in the online setting compared to the in-person setting, assessing the questions who might be suitable to become an online psychotherapist, who might train them, and what kind of education programs might be suitable in which form Data security issues assessing secure ways of communication using different technologies, also regarding secure data storage and secure online payment

## Conclusion

If trained psychotherapists choose not to participate in the new and emerging field of online psychotherapy, it seems likely that charlatans will emerge to meet the ever-growing demand, perhaps driving professional psychotherapists out of the market (37). For that reason, psychotherapists from all professional backgrounds must be properly informed about the risks and benefits of online psychotherapy if they are to make well-informed decisions and act in the best interests of their patients. Even if they decide not to offer such services themselves, they should be equipped to provide information about online psychotherapy that enables patients to make a well-considered decision about using such services.

## Author Contributions

JS and MT designed the review and developed the search strategy. JS and JM were involved in search, exclusion, and argument extraction processes. JS and MT wrote and edited the final article, which was reviewed and approved by JM.

## Conflict of Interest

The authors declare that the research was conducted in the absence of any commercial or financial relationships that could be construed as a potential conflict of interest.
