# Supplementary material for: Ethical Issues in Online Psychotherapy: A Narrative Review
Source: Front Psychiatry. 2020 Feb 11;10:993. doi: 10.3389/fpsyt.2019.00993 (PMC7026245; doi:10.3389/fpsyt.2019.00993)
Supplement: Supplementary file 1 [file DataSheet_1.docx]

Supplementary Material

# Full Search Code

**Search code for PubMed.** (((("Ethics"[Mesh]) OR (ethic OR ethics OR ethical))) AND ((("Behavior Control"[Mesh] OR "Psychiatry"[Mesh] OR "Psychology"[Mesh] OR "Mental Health Services"[Mesh] OR "Psychiatric Somatic Therapies"[Mesh] OR "Psychoanalytic Interpretation"[Mesh] OR "Psychological Techniques"[Mesh] OR "Psychotherapy"[Mesh] OR "Schizophrenic Psychology"[Mesh])) OR (psychiatry OR psychology OR psychotherapy OR psychological OR psychiatric OR “mental health“))) AND ((("Internet"[Mesh] OR "Software/therapeutic use"[Mesh] OR "Distance Counseling"[Mesh] OR "Telecommunications"[Mesh] OR "Telemedicine"[Mesh])) OR (online OR online-psychotherapy OR tele OR tele-psychotherapy OR internet OR internet-psychotherapy OR internet-based OR internet-mediated OR computer OR computer-based OR web OR web-based OR e-therapy OR e-psychotherapy OR “e-mental health” OR mhealth OR e-health OR ehealth OR teleadvice OR telehealth OR “tele-mental health” OR telepsychiatry OR telepsychology OR telemedicine OR telepsychotherapy OR teletherapy OR telecommunication OR telecommunications OR “electronically mediated mental health” OR “electronically mediated psychotherapy” OR “counseling on the internet” OR “therapy on the internet” OR “technology assisted distance counseling” OR “technology assisted distance psychotherapy” OR “clinical service via the internet” OR “clinical services via the internet” OR “clinical service online” OR “clinical services online” OR “mobile health”))

**Search code for PsycINFO.** ((DE "Ethics" OR DE "Bioethics" OR DE "Business Ethics" OR DE "Consumer Ethics" OR DE "Experimental Ethics" OR DE "Plagiarism" OR DE "Professional Ethics" ) OR ( ethic OR ethics OR ethical ) ) AND ( ( DE "Psychotherapy" OR DE "Adlerian Psychotherapy" OR DE "Adolescent Psychotherapy" OR DE "Affirmative Therapy" OR DE "Analytical Psychotherapy" OR DE "Autogenic Training" OR DE "Behavior Therapy" OR DE "Brief Psychotherapy" OR DE "Brief Relational Therapy" OR DE "Child Psychotherapy" OR DE "Client Centered Therapy" OR DE "Cognitive Behavior Therapy" OR DE "Conversion Therapy" OR DE "Eclectic Psychotherapy" OR DE "Emotion Focused Therapy" OR DE "Existential Therapy" OR DE "Experiential Psychotherapy" OR DE "Expressive Psychotherapy" OR DE "Eye Movement Desensitization Therapy" OR DE "Feminist Therapy" OR DE "Geriatric Psychotherapy" OR DE "Gestalt Therapy" OR DE "Group Psychotherapy" OR DE "Guided Imagery" OR DE "Humanistic Psychotherapy" OR DE "Hypnotherapy" OR DE "Individual Psychotherapy" OR DE "Insight Therapy" OR DE "Integrative Psychotherapy" OR DE "Interpersonal Psychotherapy" OR DE "Logotherapy" OR DE "Narrative Therapy" OR DE "Network Therapy" OR DE "Persuasion Therapy" OR DE "Primal Therapy" OR DE "Psychoanalysis" OR DE "Psychodrama" OR DE "Psychodynamic Psychotherapy" OR DE "Psychotherapeutic Counseling" OR DE "Rational Emotive Behavior Therapy" OR DE "Reality Therapy" OR DE "Relationship Therapy" OR DE "Solution Focused Therapy" OR DE "Supportive Psychotherapy" OR DE "Transactional Analysis" OR DE "Multisystemic Therapy" OR DE "Aversion Therapy" OR DE "Dialectical Behavior Therapy" OR DE "Exposure Therapy" OR DE "Implosive Therapy" OR DE "Reciprocal Inhibition Therapy" OR DE "Response Cost" OR DE "Systematic Desensitization Therapy" OR DE "Play Therapy" OR DE "Acceptance and Commitment Therapy" OR DE "Empty Chair Technique" OR DE "Age Regression (Hypnotic)" OR DE "Ericksonian Psychotherapy" OR DE "Schema Therapy" OR DE "Dream Analysis" OR DE "Self-Analysis" OR DE "Family Therapy" OR DE "Psychiatry" OR DE "Psychology" ) OR ( psychiatry OR psychology OR psychotherapy OR psychological OR psychiatric OR mental health ) ) AND ( ( DE "Online Therapy" OR DE "Computer Applications" OR DE "Cloud Computing" OR DE "Computer Assisted Design" OR DE "Computer Assisted Diagnosis" OR DE "Computer Assisted Instruction" OR DE "Computer Assisted Testing" OR DE "Computer Assisted Therapy" OR DE "Computer Simulation" OR DE "Electronic Learning" OR DE "Groupware" OR DE "Hypermedia" OR DE "Hypertext" OR DE "Computer Mediated Communication" OR DE "Blog" OR DE "Health Care Seeking Behavior" OR DE "Telecommunications Media" OR DE "Radio" OR DE "Telephone Systems" OR DE "Television" OR DE "Television Advertising" OR DE "Telemedicine" OR DE "Internet" OR DE "Electronic Communication" OR DE "Social Media" OR DE "Text Messaging" ) OR ( online OR online-psychotherapy OR tele OR tele-psychotherapy OR internet OR internet-psychotherapy OR internet-based OR internet-mediated OR computer OR computer-based OR web OR web-based OR e-therapy OR e-psychotherapy OR e-mental health OR mhealth OR e-health OR ehealth OR teleadvice OR telehealth OR tele-mental health OR telepsychiatry OR telepsychology OR telemedicine OR telepsychotherapy OR teletherapy OR telecommunication OR telecommunications OR electronically mediated mental health OR electronically mediated psychotherapy OR counseling on the internet OR therapy on the internet OR technology assisted distance counseling OR technology assisted distance psychotherapy OR clinical service via the internet OR clinical services via the internet OR clinical service online OR clinical services online OR mobile health))

**Search code for Web of Science.** ((ethic OR ethics OR ethical) AND (psychiatry OR psychology OR psychotherapy OR psychological OR psychiatric OR “mental health“) AND (online OR online-psychotherapy OR tele OR tele-psychotherapy OR internet OR internet-psychotherapy OR internet-based OR internet-mediated OR computer OR computer-based OR web OR web-based OR e-therapy OR e-psychotherapy OR “e-mental health” OR mhealth OR e-health OR ehealth OR teleadvice OR telehealth OR “tele-mental health” OR telepsychiatry OR telepsychology OR telemedicine OR telepsychotherapy OR teletherapy OR telecommunication OR telecommunications OR “electronically mediated mental health” OR “electronically mediated psychotherapy” OR “counseling on the internet” OR “therapy on the internet” OR “technology assisted distance counseling” OR “technology assisted distance psychotherapy” OR “clinical service via the internet” OR “clinical services via the internet” OR “clinical service online” OR “clinical services online” OR “mobile health”))

# Publication Selection Criteria

- Online psychotherapy or counseling by a psychiatrist, psychologist or social worker
- No layman
- No nurses
- No assessment (online diagnostic/testing)
- No supervision
- No teaching
- No online health monitoring
- No palliative care
- No forensics
- No ethical issues with online health records (databases)
- No sport psychology
- No organizational or business psychology
- No occupational therapy
- No neuropsychology (more assessment than therapy, except explicit concentration on psychotherapy of psychiatric mental health issues)
- No military psychology/psychiatry
- No spiritual care
- No art therapy, music or dance therapy
- No grief counseling
- No prevention/ rehabilitation (concentration on psychiatric mental health issues, no somatic illnesses (e.g. cancer))
- No telemedicine or telecare (only if concentration on psychiatric mental health issues)
- No gerontopsychology/psychiatry
- No therapy for intellectual disabilities
- No child or adolescent psychology/psychiatry (no school psychology)
- No group therapy
- No health chats
- No family therapy
- No couple therapy
- No student therapy (counseling on the campus; only if concentration on psychiatric mental health issues)
- No online self-care applications
- No therapy through videogames
- No online psychoeducation
- No psychotherapy by robots or bots or online programs (without “human” interaction, by computer brain interfaces; no avatars/virtual worlds/second life)
- No health websites (except direct psychotherapy over these sites)
- No ethical issues with social media
